# Silver-Assembled Silica Nanoparticles in Lateral Flow Immunoassay for Visual Inspection of Prostate-Specific Antigen

**DOI:** 10.3390/s21124099

**Published:** 2021-06-15

**Authors:** Hyung-Mo Kim, Jaehi Kim, Sungje Bock, Jaehyun An, Yun-Sik Choi, Xuan-Hung Pham, Myeong Geun Cha, Bomi Seong, Wooyeon Kim, Yoon-Hee Kim, Hobeom Song, Jung-Won Kim, Seung-min Park, Sang Hun Lee, Won-Yeop Rho, Sangchul Lee, Dae Hong Jeong, Ho-Young Lee, Bong-Hyun Jun

**Affiliations:** 1 Department of Bioscience and Biotechnology, Konkuk University, Seoul 05029, Korea; hmkim0109@konkuk.ac.kr (H.-M.K.); susia45@gmail.com (J.K.); bsj4126@konkuk.ac.kr (S.B.); ghj4067@konkuk.ac.kr (J.A.); phamricky@gmail.com (X.-H.P.); iambomi33@konkuk.ac.kr (B.S.); jgk03041@naver.com (W.K.); yoonhees@konkuk.ac.kr (Y.-H.K.); 2 Department of Chemistry Education, Seoul National University, Seoul 05029, Korea; 71388c@naver.com (Y.-S.C.); cha6614@snu.ac.kr (M.G.C.); jeongdh@snu.ac.kr (D.H.J.); 3 BioSquare Inc., Seongnam 13620, Korea; hbsong@bio-square.com (H.S.); jwkim@bio-square.com (J.-W.K.); 4 Department of Radiology, Stanford University School of Medicine, Stanford, CA 94305, USA; sp293@stanford.edu; 5 Molecular Imaging Program at Stanford, Stanford University School of Medicine, Stanford, CA 94305, USA; 6 Department of Chemical and Biological Engineering, Hanbat National University, Daejeon 34158, Korea; sanghunlee@hanbat.ac.kr; 7 School of International Engineering and Science, Jeonbuk National University, Jeonju 54896, Korea; rho7272@jbnu.ac.kr; 8 Department of Urology, Seoul National University Bundang Hospital, Seongnam 13620, Korea; slee@snubh.org; 9 Department of Nuclear Medicine, Seoul National University Bundang Hospital, Seongnam 13620, Korea

**Keywords:** silica nanoparticles, prostate-specific antigen, lateral flow immunoassay

## Abstract

Prostate-specific antigen (PSA) is the best-known biomarker for early diagnosis of prostate cancer. For prostate cancer in particular, the threshold level of PSA <4.0 ng/mL in clinical samples is an important indicator. Quick and easy visual detection of the PSA level greatly helps in early detection and treatment of prostate cancer and reducing mortality. In this study, we developed optimized silica-coated silver-assembled silica nanoparticles (SiO_2_@Ag@SiO_2_ NPs) that were applied to a visual lateral flow immunoassay (LFIA) platform for PSA detection. During synthesis, the ratio of silica NPs to silver nitrate changed, and as the synthesized NPs exhibited distinct UV spectra and colors, most optimized SiO_2_@Ag@SiO_2_ NPs showed the potential for early prostate cancer diagnosis. The PSA detection limit of our LFIA platform was 1.1 ng/mL. By applying each SiO_2_@Ag@SiO_2_ NP to the visual LFIA platform, optimized SiO_2_@Ag@SiO_2_ NPs were selected in the test strip, and clinical samples from prostate cancer patients were successfully detected as the boundaries of non-specific binding were clearly seen and the level of PSA was <4 ng/mL, thus providing an avenue for quick prostate cancer diagnosis and early treatment.

## 1. Introduction

Biomarker detection facilitates early diagnosis and monitoring of disease status, such as in cancer [1]. Cancer cells generate a variety of biomarkers, and their detection provides important information about the type and present stage of the target cancer [2]. Immunological assays have been commonly used for the detection of these biomarkers in a non- or minimally-invasive manner [3,4,5]. Typical techniques for immunological analysis include enzyme-linked immunosorbent assay (ELISA), radio immunoassay (RIA), and the fluorescent antibody (Ab) (FA) technique [6,7,8]. Among these, ELISA is a representative immunological analysis method widely used as a diagnostic tool in medicine due to its high reproducibility and strong quantitative approach [9], but it requires large amounts of samples (20–200 μL), has a lengthy analysis time (1.5–3 h), and requires expensive instrumentation for quantitative analysis [10]. An alternative to ELISA, paper-based ELISA, in which paper is used as a substrate for AB immobilization, can be performed quickly (~1 h) using a small amount of the sample (1–10 μL) and simple equipment (commercial scanners); however, a disadvantage is that it has a lower sensitivity than conventional ELISA [10,11].

Recent focus has centered on another alternative, lateral flow immunoassay (LFIA), which involves the use of a test strip. LFIA is widely used for clinical diagnosis as well as for food safety, animal health, and environmental monitoring. LFIA can be performed quickly for clinical diagnosis applications because there is no additional sedimentation or cleaning procedure during the Ab–antigen binding process. It can also be used for early diagnosis using cancer biomarkers in a variety of samples, such as saliva, urine, and serum [12,13,14], and has the advantage of being simpler and more economical than ELISA. Since early cancer diagnosis is very important for cancer treatment and can increase patient survival rates, LFIA can be applied in early cancer diagnosis to maximize survival [15].

Among the various kind of cancers, prostate cancer is the second most diagnosed cancer in the world and is the sixth leading cause of cancer-related death worldwide [16]. Prostate-specific antigen (PSA) was known to be reproducible in blood of men without biopsy of target tissue that has increased levels in prostate cancer, is detectable in the early stages of prostate cancer and was the most reliable biomarker for monitoring prognosis [17,18,19,20]. Early diagnosis of prostate cancer by PSA detection lowers the mortality rate and plays an important role in treatment [21,22]. Conventional tests for prostate cancer diagnoses using PSA are mainly performed in centralized laboratories using large-scale automated analyzers, and the associated medical costs increase due to various reasons, such as sample transport, increased waiting time, and simple management. However, these detection methods often lead to over-treatment and over-diagnosis; hence, there is a need for a method that can detect PSA quickly, accurately, and economically [19,21]. Using LFIA for prostate cancer diagnosis, PSA can be detected at low costs and in a shorter duration, increasing patient satisfaction. Traditionally, the normal PSA level that is acceptable as the basis for biopsy in phase 3 clinical studies of early prostate cancer screening is <4.0 ng/mL [20], and this serves as an important indicator for judging the presence or absence of prostate cancer via LFIA [23,24].

To analyze clinical samples in LFIA, we used a silica NP-based assembly of silver NPs (AgNPs). Among the various metal NPs, AgNPs can be synthesized as nanomaterials with the desired chemical, morphological, and optical properties by controlling the reaction conditions [25]. When these AgNPs are manufactured in assembly form, they can have added advantages in biological applications as their optical effect is maximized [26,27]. It is known that the assembly structure is capable of controlling localized surface plasmon resonance (LSPR) and shows better scattering properties than single NPs [28]. In LFIA, outcomes such as low LOD using assembly-type nanoprobes have already been demonstrated [29,30]. As such, the use of assembly-type nanoprobes is a good way to lower the LOD in LFIA, but as far as we know, PSA detection through LFIA using assembled AgNP structures has not been reported.

Here, we present an LFIA system using optimized silica-coated silver-assembled silica (SiO_2_@Ag@SiO_2_) NPs that can be detected in PSA from serum. Three types of SiO_2_@Ag@SiO_2_ NPs, capable of absorbing a wide range of wavelengths, were synthesized by precisely controlling the amount of AgNPs on the silica NPs. Absorption of a wide range of wavelengths showed improved LOD in PSA detection compared to other SiO_2_@Ag@SiO_2_ NPs. Furthermore, when clinical samples were introduced and developed, all test strip results were clearly distinguished with the naked eye compared to a negative control using SiO_2_@Ag@SiO_2_ NPs.

## 2. Materials and Methods

### 2.1. Materials

Tetraethyl orthosilicate (TEOS), (3-mercaptopropyl)trimethoxysilane (MPTS), ethylene glycol (EG), silver nitrate (AgNO_3_, 99.99%), octylamine (OA), sodium silicate solution, (3-aminopropyl)triethoxysilane (APTS), succinic anhydride, N, N-diisopropylethylamine (DIEA), N-(3-dimethylaminopropyl)-N′-ethylcarbodiimide hydrochloride (EDC), N-hydroxysulfosuccinimide sodium salt (Sulfo-NHS), 2-(N-morpholino)ethanesulfonic acid (MES) hydrate, phosphate-buffered saline (PBS, pH 7.4), TWEEN^®^ 20, 11-mercaptoundecanoic acid (11-MUA), and ethanolamine were purchased from Sigma–Aldrich (St. Louis, MO, USA). Ethyl alcohol (EtOH, 99.9%), aqueous ammonium hydroxide (NH_4_OH, 27%), and 1-methyl-2-pyrrolidinone (NMP) were purchased from Daejung (Siheung, Korea). Amino polyethylene glycol (PEG) Acid (NH_2_–PEG–COOH, MW = 600 Da) was purchased from Nanocs Inc. (New York, NY, USA). The backing card, nitrocellulose (NC) membrane, absorbent pad, cassette, mouse monoclonal anti-PSA Ab (14,801 and 14,803), and goat anti-rabbit IgG Ab were purchased from Bore Da Biotech Co. Ltd. (Seongnam, Korea).

### 2.2. Synthesis of SiO_2_@Ag@SiO_2_ NPs

SiO_2_ NPs were synthesized using a modified Stöber method [25]. A solution containing TEOS (1.6 mL), NH_4_OH (3 mL), and absolute EtOH (40 mL) was stirred for 20 h at 25 °C. The solution was washed several times with absolute EtOH by centrifugation at 8885 rcf for 10 min repeatedly. To introduce thiol groups, SiO_2_ NPs (200 mg) were mixed with absolute EtOH (4 mL), MPTS (200 μL), and NH_4_OH (40 μL) for 12 h at 25 °C. The solution was washed several times with absolute EtOH by centrifugation at 8885 rcf for 10 min. Following thiol-functionalization, AgNPs were introduced onto the surface of SiO_2_ NPs by using a modified polyol process. In this process, each SiO_2_@Ag NP was introduced by using different amounts of AgNO_3_: 2.6 mg AgNO_3_ per 1 mg SiO_2_ NPs, 0.9 mg AgNO_3_ per 1 mg SiO_2_ NPs, and 0.5 mg AgNO_3_ per 1 mg SiO_2_ NPs with PVP (MW 40,000) in 50 mL EG. Then, 41.4 μL OA was added sequentially to each solution, and the solutions were stirred for 1 h at 25 °C. Each solution was washed several times with absolute EtOH by centrifugation at 8885 rcf for 10 min. Silica shells were introduced to each SiO_2_@Ag NP via the modified silica coating method [31]. Each SiO_2_@Ag NP (1 mg) was dispersed in 1 mL absolute EtOH with 0.15 mM 11-MUA and stirred for 1 h at 25 °C. Then, each SiO_2_@Ag NP was dispersed in 15 mL aqueous sodium silicate solution (0.036 wt%) and stirred for 15 h at 25 °C. Subsequently, 60 mL absolute EtOH was added to each solution and stirred for 3 h at 25 °C to form a thin silica shell. After 3 h, 30 μL TEOS and aqueous ammonium hydroxide (28–30%, 250 μL) were added to each solution and stirred for 24 h at 25 °C. The resulting SiO_2_@Ag@SiO_2_ NP_2.6_, SiO_2_@Ag@SiO_2_ NP_0.9_, and SiO_2_@Ag@SiO_2_ NP_0.5_ were washed several times with absolute EtOH by centrifugation at 8885 rcf for 10 min.

### 2.3. Conjugation for Ab

Ab conjugation were explained as a schematic illustration (Supplementary Materials: Figure S1). For the introduction of amine groups to the surface, each SiO_2_@Ag@SiO_2_ NP (1 mg) was mixed with APTS (10 μL) and NH_4_OH (10 μL) for 1 h at 25 °C. The mixture was washed several times with NMP by centrifugation at 15,928 rcf for 10 min. To introduce carboxyl groups onto the surface of amine-functionalized SiO_2_@Ag@SiO_2_ NPs, they were dispersed in NMP (500 μL consisting of 1.75 mg succinic anhydride), and the mixture was added to DIEA (3.05 μL) and stirred for 2 h at 25 °C. The mixture was washed several times with deionized water (DW) by centrifugation at 15,928 rcf for 10 min, followed by redispersion in MES (50 mM). Carboxyl-functionalized SiO_2_@Ag@SiO_2_ NPs were added to EDC (2 mg) and sulfo-NHS (2 mg), and the mixture was stirred for 30 min at 25 °C. The supernatant was removed, followed by dispersion in MES (50 mM). The activation group-functionalized SiO_2_@Ag@SiO_2_ NPs were added to NH_2_-PEG_600_-COOH (1.6 mM), and the mixture was stirred for 2 h at 25 °C. For surface blocking, SiO_2_@Ag@SiO_2_ NP–PEG-COOH was added to ethanolamine (3.2 μL) and stirred for 30 min at 25 °C. The mixture was washed several times with DW by centrifugation at 15,928 rcf for 10 min and redispersed in MES (50 mM). SiO_2_@Ag@SiO_2_ NP–SNs–PEG-COOH was added to EDC (2 mg) and sulfo-NHS (2 mg) and stirred for 30 min at 25 °C. The supernatant was removed and dispersed in MES (50 mM). The activation group-supplemented SiO_2_@Ag@SiO_2_ NP–SNs–PEG-COOH was added to anti-PSA Ab (14,803) and stirred for 2 h at 25 °C. After centrifugation, SiO_2_@Ag@SiO_2_ NP–SNs–PEG-PSA Ab was added to ethanolamine (3.2 μL) and stirred for 30 min at 25 °C. The mixture was washed several times with 0.5% bovine serum albumin (BSA) by centrifugation at 15,928 rcf for 10 min and redispersed in 0.5% BSA.

### 2.4. Preparation of Test Strips

The test strip consisted of a backing card, NC membrane, and absorbent pad. After assembling the NC membrane on the backing card, the test line was sprayed with anti-PSA Ab (14,801, 1 mg/mL) using a dispenser, and the control line was sprayed with goat anti-rabbit IgG Ab (1 mg/mL). The assembly was dried for 2 h. Thereafter, 0.1% BSA was applied to the assembly and dried for at least a day. Finally, the absorbent pad was assembled on the backing card. After cutting the strip to a length of 0.4 cm, the test strip preparation was completed.

### 2.5. Analysis of Colored Band of the Test Line on the Test Strip

To measure the intensity of the colored band of the test line on the test strip, each strip was captured as an 8-byte image using ImageQuant LAS-4000 (GE Healthcare, Chicago, IL, USA) [32]. Captured images were analyzed using the ImageJ software (ver. 1.53a, National Institutes of Health, Bethesda, MD, USA).

### 2.6. Characterization of SiO_2_@Ag@SiO_2_ NPs

Transmission electron microscopy (TEM) images were obtained using a LIBRA 120 (Carl Zeiss, Oberkochen, Germany). UV–vis extinction spectra were obtained with an Optigen 2120 UV spectrophotometer (Mecasys, Daejeon, Korea).

## 3. Results and Discussion

### 3.1. Synthesis of Each SiO_2_@Ag@SiO_2_ NP

**Figure 1:**
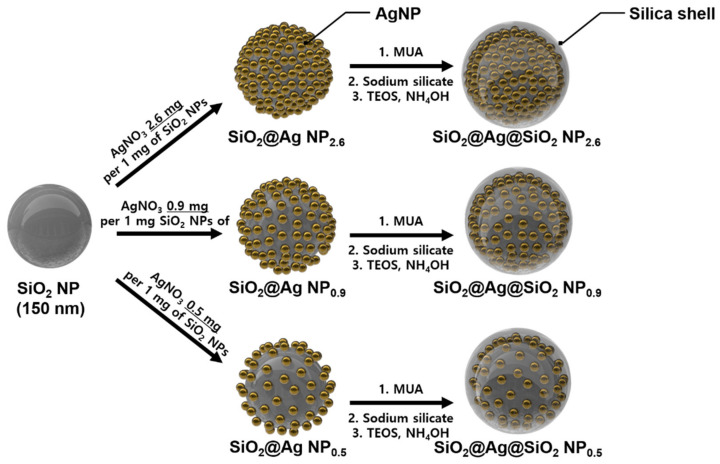
Illustration of a typical preparation of silver assembly silica nanoprobes (SiO_2_@Ag@SiO_2_ NPs).

**Figure 2:**
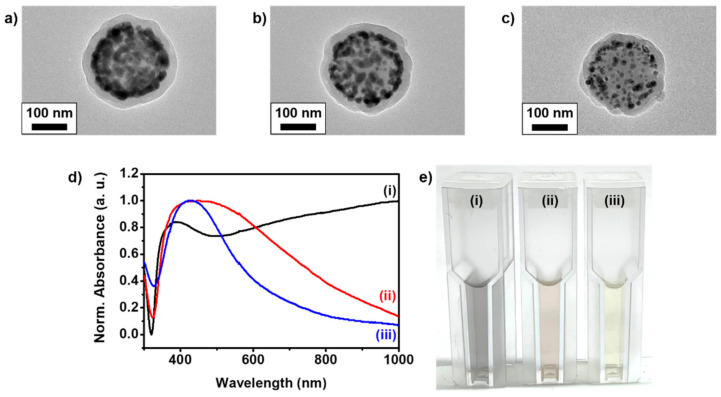
Transmission electron microscopy (TEM) images of (**a**) SiO_2_@Ag@SiO_2_ NP_2.6_, (**b**) SiO_2_@Ag@SiO_2_ NP_0.9_, and (**c**) SiO_2_@Ag@SiO_2_ NP_0.5_. (**d**) UV–Vis absorption spectra and (**e**) actual color image of (i) SiO_2_@Ag@SiO_2_ NP_2.6_, (ii) SiO_2_@Ag@SiO_2_ NP_0.9_, and (iii) SiO_2_@Ag@SiO_2_ NP_0.5_ in EtOH.

The synthesis procedure for each SiO_2_@Ag@SiO_2_ NP is shown in Figure 1 [26,33]. In the process of assembling AgNPs on SiO_2_ NPs, we attempted to evaluate optimized SiO_2_@Ag@SiO_2_ NPs for detecting specific PSA concentrations on an LFIA platform based on the pattern formed with the introduction of AgNPs. The nanoprobes used in an optimized LFIA can affect the antigen–Ab interaction and LOD [34,35]. Additionally, SiO_2_ NPs were synthesized by using the sol–gel process based on the Stöber method. They were analyzed via TEM and were found to be spherical, monodispersed, and uniformly sized (167 ± 3.5 nm, *n* = 30; Figure S2). Then, a thiol group was introduced on the surface of each SiO_2_ NP via a reaction with MPTS. For the fabrication of the assembled AgNPs and SiO_2_ NPs (SiO_2_@Ag NPs), AgNPs were allowed to develop on the surface of SiO_2_ NPs, which introduced the thiol group. Octylamine and ethylene glycol have important roles in the nucleation and growth of AgNPs on the surface of SiO_2_ NPs [36], and silver ions were effectively reduced on the surface of SiO_2_ NPs because of the interaction between the thiol groups of MPTS and the silver ions, producing AgNPs. Simultaneously, it was confirmed that three types of SiO_2_@Ag NPs, named according to their AgNO_3_ and SiO_2_ NP ratios (SiO_2_@Ag NP_2.6_, SiO_2_@Ag NP_0.9_, and SiO_2_@Ag NP_0.5_) were synthesized by adjusting the amount of AgNO_3_ (Figure S3). Subsequently, 11-MUA, which has both terminal thiol and carboxyl groups, was introduced to the surface of the particles to functionalize the surface of AgNPs and facilitate an additional silica coating of the surface [31]. To prevent aggregation and facilitate additional surface modifications, each SiO_2_@Ag NP was encapsulated in an outer silica shell, and three types of silica shell-coated SiO_2_@Ag NPs (SiO_2_@Ag@SiO_2_ NP_2.6_, SiO_2_@Ag@SiO_2_ NP_0.9_, and SiO_2_@Ag@SiO_2_ NP_0.5_) were finally synthesized. TEM images (Figure 2a–c) show that all SiO_2_@Ag@SiO_2_ NPs were well encapsulated within the silica shell. The plasmon resonance spectra of all SiO_2_@Ag@SiO_2_ NP solutions were analyzed by UV–vis spectroscopy (Figure 2d). SiO_2_@Ag@SiO_2_ NPs produced by controlling the amount of AgNO_3_ were red-shifted as the amount of SiO_2_ NPs was reduced, indicating the induced surface plasmon resonance shift, which agreed with a previous study [37]. Moreover, each synthesized SiO_2_@Ag@SiO_2_ NP was visually compared in ethyl alcohol (Figure 2e). Their color, which was highly dependent on synthesized AgNPs, gradually changed from yellow to dark gray as the amount of SiO_2_ NPs decreased. By controlling the amount of AgNO_3_ during the synthesis of SiO_2_@Ag@SiO_2_ NPs, we successfully synthesized three types of SiO_2_@Ag@SiO_2_ NPs and confirmed that the synthesized particles had plasmon resonance spectra.

### 3.2. Measurement of the Scattering Effect in the NC Membrane on the Test Strip

**Figure 3:**
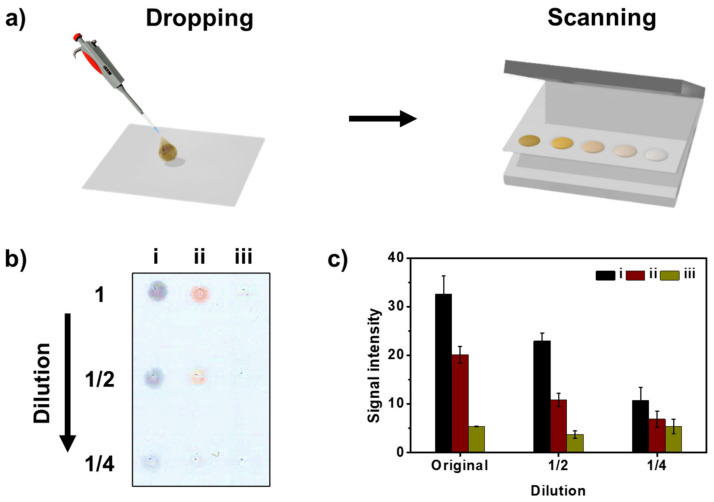
(**a**) Spotting of SiO_2_@Ag@SiO_2_ NPs onto the membrane and data analysis. (**b**) Scanned color image of the spots formed on the nitrocellulose (NC) membrane with 0.5 μL of suspensions at different concentrations (1/2 dilutions) and (**c**) intensity of corresponding colored spots on the NC membrane of (i) SiO_2_@Ag@SiO_2_ NP_2.6_, (ii) SiO_2_@Ag@SiO_2_ NP_0.9_, and (iii) SiO_2_@Ag@SiO_2_ NP_0.5_. Error bars represent the standard deviation of the mean over the three batches of measurements of analytes.

Before application of each SiO_2_@Ag@SiO_2_ NP to LFIA, we confirmed the scattering effect of light in the NC membrane in the test strip. Normally, the colored band representing the result of the NC membrane absorbs and scatters light according to the plasmon properties of the probe [38]. Referring to the previous study, a series of three consecutive 1/2 dilutions of SiO_2_@Ag@SiO_2_ NP_2.6_, SiO_2_@Ag@SiO_2_ NP_0.9_, and SiO_2_@Ag@SiO_2_ NP_0.5_ were used [39]. The prepared samples were dropped by spotting on the NC membrane, and visibility by scattering of color spots according to NP dilution was confirmed. First, 0.5 μL of each sample was dropped onto the NC membrane and dried, and then the NC membrane was scanned through a commercial scanner (L3150, Epson, Suwa, Japan) (Figure 3a). Figure 3b shows an image of colored spots obtained by applying a double dilution (top to bottom). Visual examination showed that the colored spot faded as each sample was diluted. When all probes were diluted to ¼ of the initial concentration, only the color intensity of SiO_2_@Ag@SiO_2_ NP_2.6_ was observed. To compare the color intensities among all particles, the intensity of each colored spot was measured using the ImageJ software (Figure 3c). SiO_2_@Ag@SiO_2_ NP_2.6_ had the brightest spot when compared to other samples. The different scattering effects shown based on conditions in the actual NC membrane indicate the possibility of detecting various biomarkers with different diagnostic criteria by using particles under different conditions. Unlike the single AuNP, AgNPs on the surface of fabricated particles were located close to each other. In particular, in the case of SiO_2_@Ag@SiO_2_ NP_2.6_, AgNPs were too closely embedded, so they were attached with a distance of a few nanometers. Because this distance of a few nanometers can enhance the LSPR effect, SiO_2_@Ag@SiO_2_ NP_2.6_ showed the highest signal intensity among the fabricated particles.

### 3.3. Applications of SiO_2_@Ag@SiO_2_ NPs as Probe in LFIA

**Figure 4:**
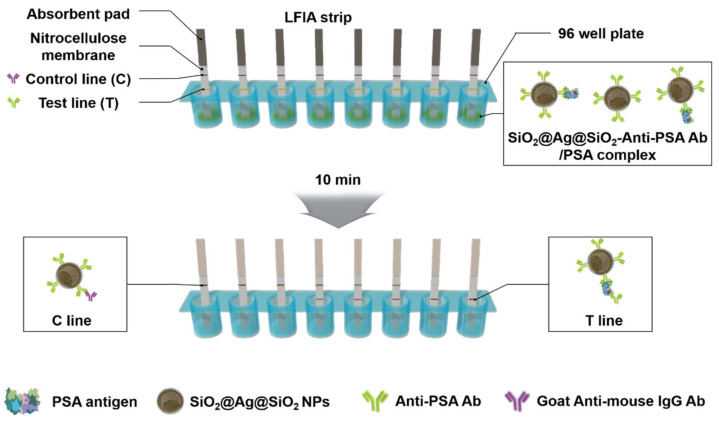
Schematic of the lateral flow immunoassay dipsticks.

To detect PSA in LFIA via the dipstick method, as schematically illustrated in Figure 4, we used a test strip consisting of an NC membrane and an absorbent pad on the backing card. In the NC membrane, anti-PSA Ab (primary Ab; 14,801) was dispensed onto the test line, and goat anti-rabbit IgG (secondary Ab) was dispensed onto the control line. For application of SiO_2_@Ag@SiO_2_ NPs, the surfaces of all SiO_2_@Ag@SiO_2_ NPs were conjugated with anti-PSA Ab (detection Ab; 14,803) using the well-known EDC/sulfo-NHS coupling reaction [40]. Here, the surfaces of all SiO_2_@Ag@SiO_2_ NPs were first conjugated with NH_2_-PEG_600_-COOH to prevent aggregation and then with anti-PSA Ab (14,803). To develop the NPs of corresponding concentration, each SiO_2_@Ag@SiO_2_ NP (3 μg) was mixed with the same amount of PSA in a 96-well plate. Then, the test strip was dipped into the corresponding well and developed, and the results were confirmed by a change in the intensity of the colored band of each test line in the test strip.

**Figure 5:**
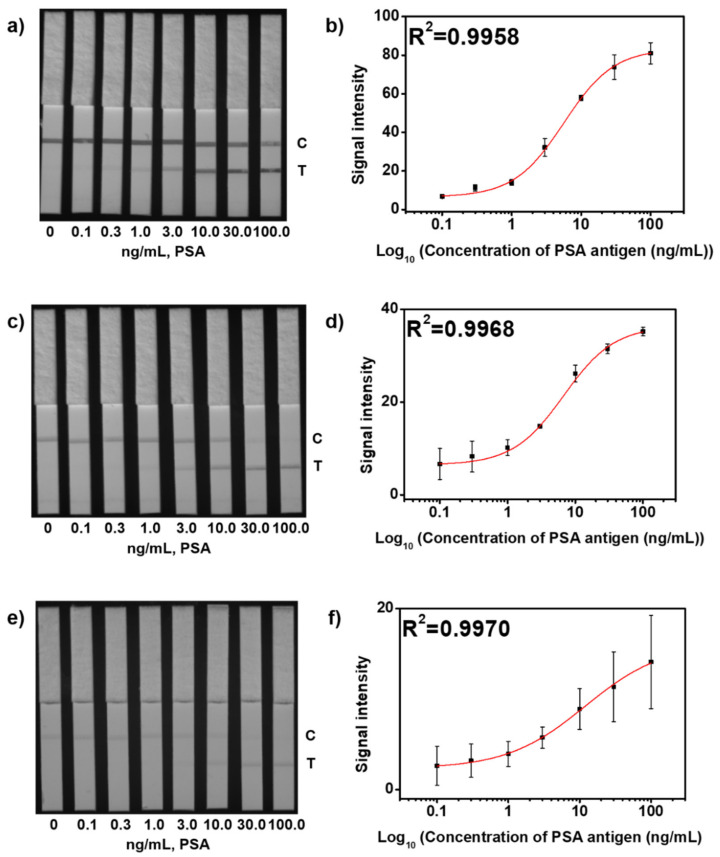
Detection of PSA antigen with various concentrations (0, 0.1, 0.3, 1.0, 3.0, 10.0, 30.0 and 100.0 ng/mL) with each SiO_2_@Ag@SiO_2_ NP in LFIA. (**a**) An 8-byte image captured using ImageQuant LAS-4000 of (**a**) SiO_2_@Ag@SiO_2_ NP_2.6_, (**c**) SiO_2_@Ag@SiO_2_ NP_0.9_, and (**e**) SiO_2_@Ag@SiO_2_ NP_0.5_. Intensity of the corresponding colored band compared to the test line on the test strip of (**b**) SiO_2_@Ag@SiO_2_ NP_2.6_, (**d**) SiO_2_@Ag@SiO_2_ NP_0.9_, and (**f**) SiO_2_@Ag@SiO_2_ NP_0.5_. Error bars represent the standard deviation of the mean over the three batches of measurements of analytes.

To observe the change in intensity of the colored band of the test line on the test strip based on the amount of PSA antigen with each SiO_2_@Ag@SiO_2_ NP, test strips were dipped in each corresponding well that contained a complex of a different concentration of PSA (0–100.0 ng/mL) with each SiO_2_@Ag@SiO_2_ NP. After 10 min, the result from each test strip was confirmed. First, non-specific binding was not observed in all test strips with 0 ng/mL developed (Figure 5a,c,e and Figure S3). The visual appearance of the colored band of the test line for each test strip was different at low concentrations. Visualization was possible for the colored band of the test line in the test strip using SiO_2_@Ag@SiO_2_ NP_2.6_ at 1.0 ng/mL, SiO_2_@Ag@SiO_2_ NP_0.9_ at 3.0 ng/mL, and SiO_2_@Ag@SiO_2_ NP_0.5_ at 10.0 ng/mL, and they could all be observed with the naked eye. When the LOD of the colored band in the test line for each probe was calculated using a logistic curve, the LOD of the test strip with SiO_2_@Ag@SiO_2_ NP_2.6_ was 1.1 ng/mL, SiO_2_@Ag@SiO_2_ NP_0.9_ was 3.0 ng/mL, and SiO_2_@Ag@SiO_2_ NP_0.5_ was 6.5 ng/mL (Figure 5b,d,f). This shows that using SiO_2_@Ag@SiO_2_ NP_2.6_ as a probe in LFIA can lower the LOD effectively.

In addition, we verified reproducibility and selectivity [41]. First, in a total of 10 batches of test strips, SiO_2_@Ag@SiO_2_ NP_2.6_ was used as a probe, and when 100 ng/mL of PSA was developed, 95.8% of reproducibility was shown, indicating excellent reproducibility (Figure S4a). In addition, when α-fetoprotein (AFP) and newborn calf serum (NCS) were developed, only PSA was selectively detected (Figure S4b).

### 3.4. Detection in Clinical Samples

**Figure 6:**
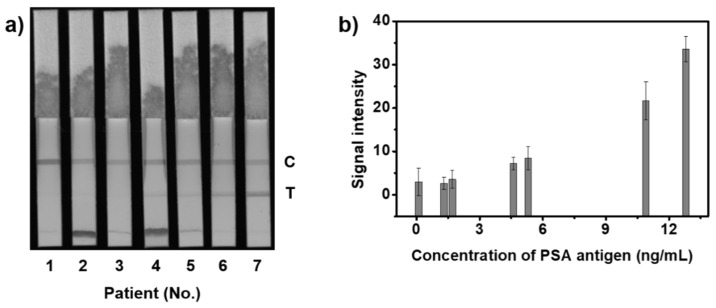
Results using clinical samples with SiO_2_@Ag@SiO_2_ NP_2.6_ in LFIA. (**a**) An 8-byte image captured using ImageQuant LAS-4000. (**b**) Intensity of the corresponding colored band compared to the test line on the test strip. Error bars represent the standard deviation of the mean over the three batches of measurements of analytes.

Based on the results obtained from the analysis of the change in color intensity with the amount of PSA, we conducted LFIA using newly constructed NPs. Among these, SiO_2_@Ag@SiO_2_ NP_2.6_ was employed as a probe for application to clinical samples in LFIA, and all clinical samples were collected from men in their 20s and 50s. Thus, we selected clinical samples spiked with PSA concentrations <0.1 ng/mL (i), negative control, 1.3 ng/mL (ii), 1.7 ng/mL (iii), 4.6 ng/mL (iv), 5.3 ng/mL (v), 10.9 ng/mL (vi), and 12.8 ng/mL (vii). In Figure 6a and Figure S5, the colored bands of the test line on the test strip that were classified as early and late stages clearly differed from those of the negative control, and the visual appearances of clinical samples i and ii, which were classified as normal, were similar to the negative control. Moreover, the intensities of normal clinical samples and negative control were similar, and the intensity of each clinical sample was slightly different in each section (Figure 6b). Therefore, these results indicate that the LFIA platform using SiO_2_@Ag@SiO_2_ NP_2.6_ as a probe is potentially useful for early prostate cancer diagnosis.

## 4. Conclusions

We fabricated an LFIA platform that can detect PSA at low concentrations and on which PSA levels in clinical samples can be observed with an optimized SiO_2_@Ag@SiO_2_ NP probe. For optimal probe selection, SiO_2_@Ag@SiO_2_ NP_2.6_, SiO_2_@Ag@SiO_2_ NP_0.9_, and SiO_2_@Ag@SiO_2_ NP_0.5_ probes that exhibited distinct color and plasmon resonance spectra were synthesized by elaborately introducing AgNPs onto silica NPs. In addition, results from the application of SiO_2_@Ag@SiO_2_ NPs to the LFIA platform could be confirmed within 10 min, and the change in intensity of the colored band of the test line on the test strip based on the amount of PSA antigen showed that the best probe, SiO_2_@Ag@SiO_2_ NP_2.6_, lowered the LOD approximately two and a half times more than conventional colloid AuNPs used in LFIA. This LFIA platform using SiO_2_@Ag@SiO_2_ NP_10_ as a probe provided easy visual observation of the PSA levels unaided by an additional device, demonstrating the possibility for similar detection of other target proteins.

## Data Availability

The data presented in this study are available on request from the corresponding author.

## Supplementary Materials

The following are available online at https://www.mdpi.com/article/10.3390/s21124099/s1, Figure S1: Schematic illustration of surface modification and conjugation of anti-PSA antibody onto the surface of fabricated particles. Figure S2: Transmission electron microscopy (TEM) images of silica nanoparticles (SiO_2_ NPs). Figure S3: TEM images of each SiO_2_@Ag NPs. (a) SiO_2_ NP@Ag NP_2.6_, (b) SiO_2_@Ag NP_0.9_, and (c) SiO_2_@Ag NP_0.5_. Figure S4: Digital photographic images of each test strip in Figure 5. (a) SiO_2_@Ag@SiO_2_ NP_2.6_, (b) SiO_2_@Ag@SiO_2_ NP_0.9_, and (c) SiO_2_@Ag@SiO_2_ NP_0.5_. Figure S5: Reproducibility and selectivity test using SiO_2_@Ag@SiO_2_ NP_2.6_. (a) Test of reproducibility using SiO_2_@Ag@SiO_2_ NP_2.6_ as a signal reporter with PSA of 100 ng/mL in LFIA. (i) Color images and (ii) measurement of signal intensity. (b) Test of Selectivity using SiO_2_@Ag@SiO_2_ NP_2.6_ as a signal reporter with Color images of (i) PSA of 100 ng/mL, (ii) α-fetoprotein (AFP) 100 ng/mL, (iii) newborn calf serum (NCS) in LFIA, and (iv) measurement of signal intensity. Figure S6: The digital photographic image of each test strip in Figure 6.
