# Supplementary material for: Silver-Assembled Silica Nanoparticles in Lateral Flow Immunoassay for Visual Inspection of Prostate-Specific Antigen
Source: Sensors (Basel). 2021 Jun 15;21(12):4099. doi: 10.3390/s21124099 (PMC8232291; doi:10.3390/s21124099)
Supplement: Supplementary file 1 [file sensors-21-04099-s001.zip › sensors-1230982-supplementary.pdf]

Silver-Assembled Silica Nanoparticles in Lateral-Flow Immunoassay for Visual Inspection of Prostate-Specific Antigen

Hyung-Mo Kim,[1] Jaehi Kim,[1] Sungje Bock,[1] Jaehyun An,[1] Yun-Sik Choi,[2] Xuan-Hung Pham,[1] Myeong Geun Cha,[2] Bomi Seong,[1] Wooyeon Kim,[1] Yoon-Hee Kim,[1] Hobeom Song,[3] Jung-Won Kim,[3] Seung-min Park,[4,5] Sang Hun Lee,[6] Won-Yeop Rho,[7] Sangchul Lee,[8] Dae Hong Jeong,[2] Ho-Young Lee[9]* and Bong-Hyun Jun[1]*

[1]Department of Bioscience and Biotechnology, Konkuk University, Seoul, Korea; hmkim0109@konkuk.ac.kr (H.-M.K.); susia45@gmail.com (J.K.); bsj4126@konkuk.ac.kr (S.B.); ghj4067@konkuk.ac.kr (J.A.); phamricky@gmail.com (X.-H.P.); iambomi33@konkuk.ac.kr (B.S.); jgk03041@naver.com (W.K.); yoonhees@konkuk.ac.kr (Y.-H.K.); bjun@konkuk.ac.kr (B.-H.J.)
[2]Department of Chemistry Education, Seoul National University, Seoul, Korea; 71388c@naver.com (Y.-S.C.); cha6614@snu.ac.kr (M.G.C.); jeongdh@snu.ac.kr (D.H.J.)
[3]BioSquare Inc., Seongnam, Korea; hbsong@bio-square.com (H.S.); jwkim@bio-square.com (J.-W.K.)
[4]Department of Radiology, Stanford University School of Medicine, Stanford, CA, USA; sp293@stanford.edu (S.-m.P.)
[5]Molecular Imaging Program at Stanford, Stanford University School of Medicine, Stanford, CA, USA
[6]Department of Chemical and Biological Engineering, Hanbat National University, Daejeon, Korea; sanghunlee@hanbat.ac.kr (S.H.L.)
[7]School of International Engineering and Science, Jeonbuk National University, Jeonju, Korea; rho7272@jbnu.ac.kr (W.-Y.R.)
[8]Department of Urology, Seoul National University Bundang Hospital, Seongnam, Korea; slee@snu.ac.kr (S.L.)
[9]Department of Nuclear Medicine, Seoul National University Bundang Hospital, Seongnam, Korea; debobkr@gmail.com (H.-Y.L.)
*Correspondence: debobkr@gmail.com; Tel.: +82-31-787-2938 (H.-Y.L.); bjun@konkuk.ac.kr; Tel.: +82-2-450-0521 (B.-H.J.)

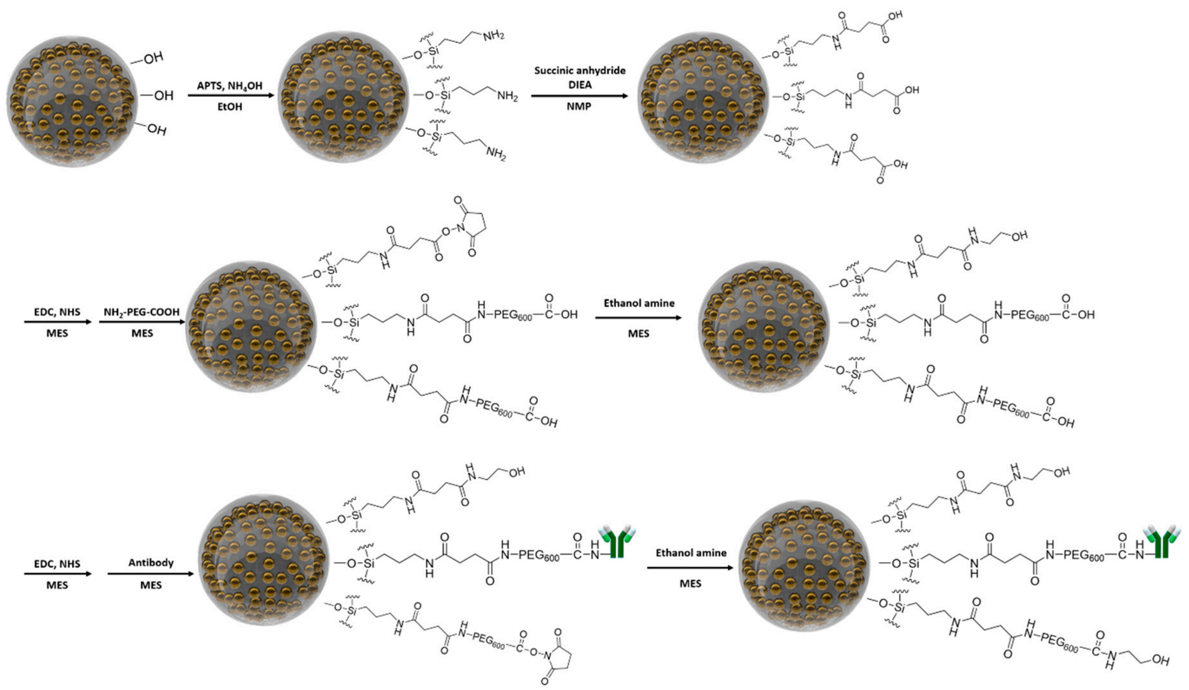

Figure S1. Schematic illustration of surface modification and conjugation of anti-PSA antibody onto the surface of fabricated particles.

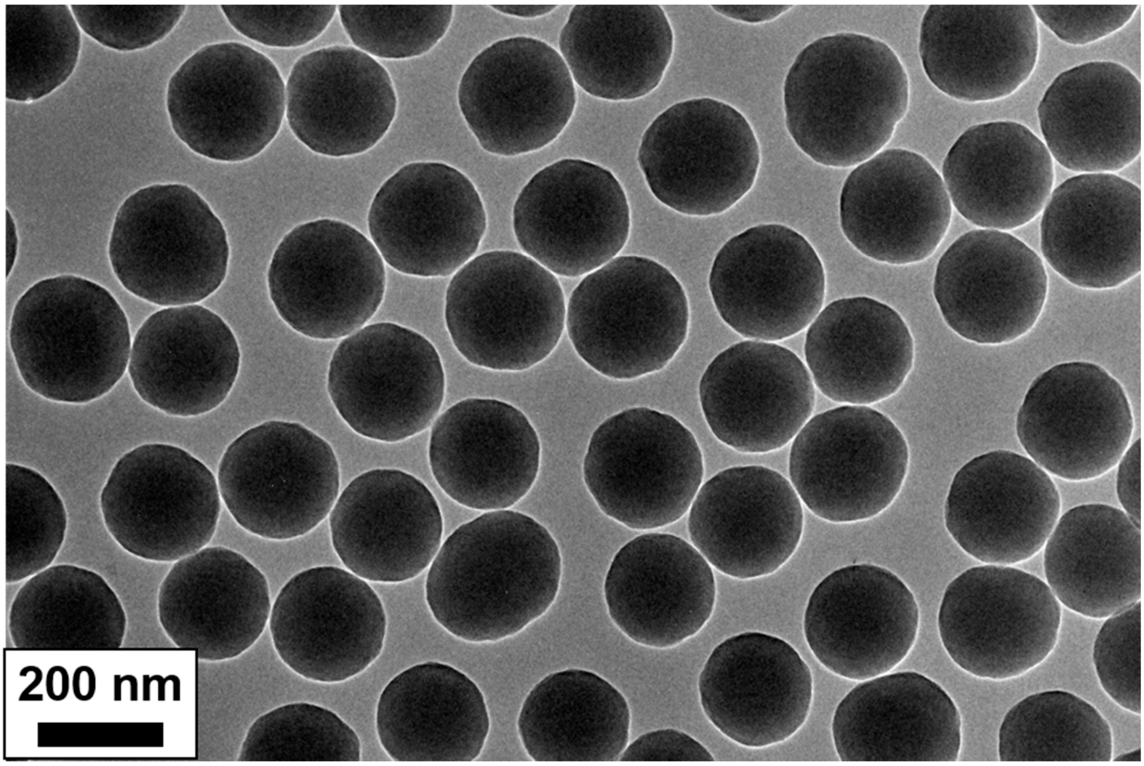

Figure S2. Transmission electron microscopy (TEM) images of silica nanoparticles (SiO_2 NPs).

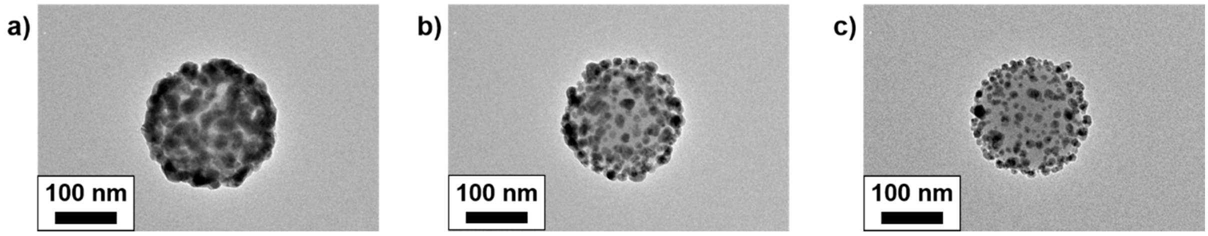

Figure S3. Transmission electron microscopy (TEM) images of each SiO_2@Ag NPs. (a) SiO_2 NP@Ag $NP_{2.6}$, (b) SiO_2@Ag $NP_{0.9}$, and (c) SiO_2@Ag $NP_{0.5}$.

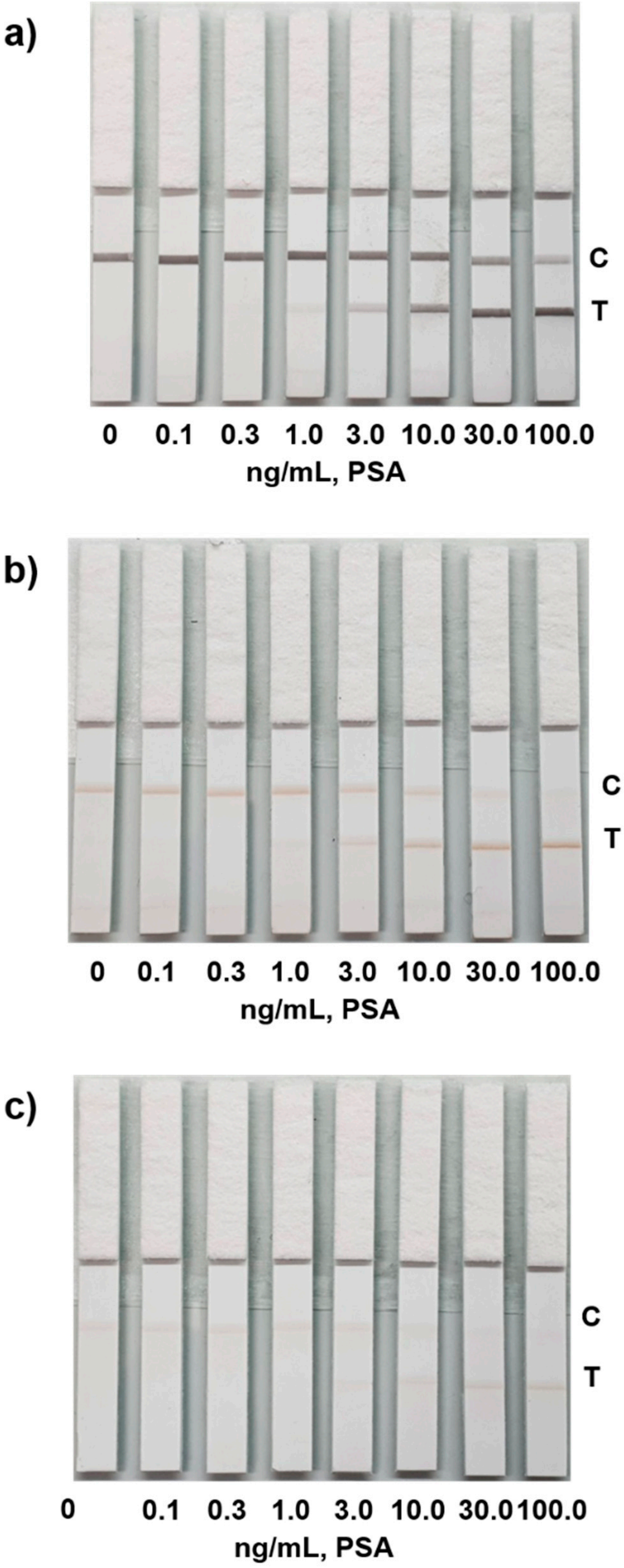

Figure S4. The digital photographic image of each test strips in Figure 5. (a) SiO_2@Ag@SiO_2 $NP_{2.6}$, (b) SiO_2@Ag@SiO_2 $NP_{0.9}$ and (c) SiO_2@Ag@SiO_2 $NP_{0.5}$.

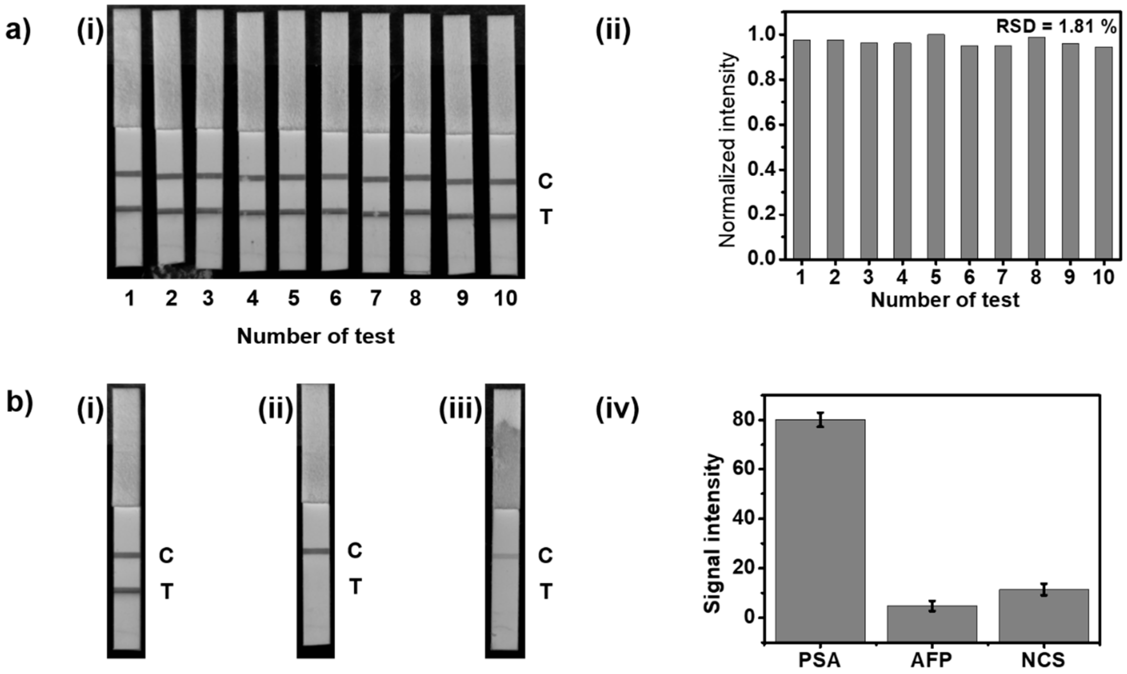

Figure S5. (a) Test of reproducibility using $SiO_2@Ag@SiO_2$ $NP_{2.6}$ as a signal reporter with PSA of 100 ng/mL in LFIA. i) Color images and ii) measurement of signal intensity. b) Test of Selectivity using $SiO_2@Ag@SiO_2$ $NP_{2.6}$ as a signal reporter with Color images of (i) PSA of 100 ng/mL, (ii) α-fetoprotein (AFP) 100 ng/mL, (iii) newborn calf serum (NCS) in LFIA, and (iv) measurement of signal intensity.

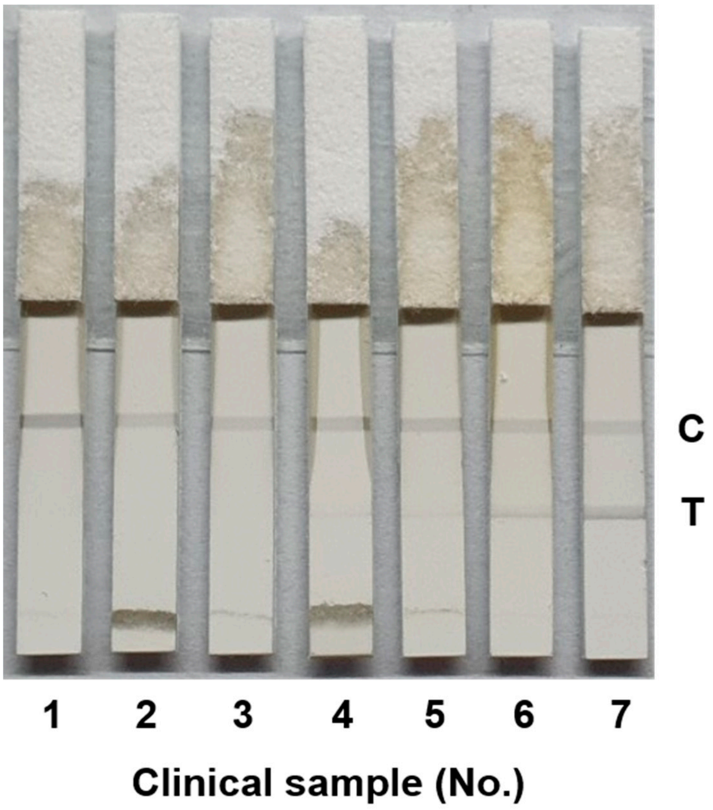

Figure S6. The digital photographic image of each test strips in Figure 6.
